# The Cuspid Teeth

**Published:** 1904-07-15

**Authors:** A. O. Hunt


### THE CUSPID TEETH.

BY A. O. HUNT, D.D.S.

A gentleman said to me that the cuspid tooth was the only tooth in the jaw that finds its proper place in the jaw; that is, that it is the least likely to erupt out of place in the jaw. This is true, and there are many reasons for it. The cuspids must come into position on account of the position that they occupy, starting from near the side of the nose coming down into position between the elevator of the lip and the elevator of the nose, and in a groove with one of these muscles on either side; it can't get out of its position aside from the natural tendency that all teeth have to go into a normal position. That is particularly true of the cuspids. In nearly all cases of irregularities you will find that the laterals or central incisors may be thrust out of position, but the cuspids in their efforts to grow in normal position will force all the other teeth out of position. Now another reason which to my mind is a most prominent one for preserving the cuspids: Running over the canine eminence are the muscles of expression. No muscle of expression can move without they all move, and when any of them move the motion is over this eminence, just the same as a certain number of ropes run through a pulley. Now the moment you lose the cuspids, just as soon as they are gone, something is gone which controls the expression of the whole face. Sometime when you are extracting a cuspid tooth, or have to do it, watch the change in the face just the moment you remove that tooth from its position. Now in making artificial dentures of all kinds we have endeavored always to bring back to the mouth that restoration of the canine eminence. We can't do it only to a limited extent, because the eminence extends completely up under the alæ of the nose. There is nothing to carry it as high as that, and in artificial dentures especially we can't carry it much higher than is commonly done. I often illustrate this by the story of the man building a house. He wanted to raise the wall, foundation wall, and still he wanted the house to look well. He went to a contractor and asked him how high he should build the wall. He said, "How high do you design building it?" "Well," he said, "I thought if it stood about three feet above the surface it would be high enough." "Well," he says, "when you decide on the height you want, then raise it a foot and your house will look better." It is just so in this—in trying to restore this canine eminence. When you have it just as it should be, carry it up a little farther. It is an impossibility for us to restore completely the expression of the face, because we cannot restore this canine eminence, and the cuspid teeth and every tooth in the mouth every means should be resorted to keep them intact as they are through life if possible.—*American Journal.*

